# Myotendinous asymmetries derived from the prolonged practice of badminton in professional players

**DOI:** 10.1371/journal.pone.0222190

**Published:** 2019-09-10

**Authors:** Alfredo Bravo-Sánchez, Pablo Abián, Fernando Jiménez, Javier Abián-Vicén

**Affiliations:** 1 Performance and Sport Rehabilitation Laboratory, Faculty of Sport Sciences, University of Castilla-La Mancha, Toledo, Spain; 2 Faculty of Humanities and Social Sciences, Comillas Pontifical University, Madrid, Spain; University of Brasilia, BRAZIL

## Abstract

**Background:**

The continued practice of a sport linked to the unilateral predominance of the dominant side can provoke chronic asymmetric adaptations in the myotendinous structure and mechanical properties. Objectives: The main purpose was to determine whether asymmetry between the preferred and non-preferred lower limb is present in the lower limb tendon structure, muscle architecture and stiffness values of professional badminton players.

**Methods:**

Sixteen male professional badminton players (age = 24.1 ± 6.7 years; body height = 177.90 ± 7.53 cm) participated in this study. The muscle architecture of the vastus lateralis (VL), medial gastrocnemius (MG) and lateral gastrocnemius (LG) and the structure of patellar and Achilles tendons were measured in the dominant and non-dominant lower limb with ultrasonography. Stiffness was also measured at the same points with a hand-held MyotonPro. Significant differences between the dominant and non-dominant lower limb were determined using Student’s t test for related samples.

**Results:**

Bilateral differences were observed for thickness, width and cross-sectional area (CSA) in both tendons showing higher values for the dominant side: patellar tendon CSA (2.02 ± 0.64 vs. 1.51 ± 0.42 cm^2^; p < 0.05) and Achilles tendon CSA (1.12 ± 0.18 vs. 0.92 ± 0.28 cm^2^; p < 0.05). No significant differences were observed in muscle architecture and myotonic variables between the dominant and non-dominant lower limb.

**Conclusions:**

The prolonged practice of badminton caused asymmetries in the CSA, width and thickness of the patellar and Achilles tendon between the dominant and non-dominant lower limbs. No bilateral differences were found in the muscle architecture of VL, MG and LG or in the stiffness of any muscle or tendon analyzed.

## Introduction

Badminton is a non-contact sport characterized by pronounced laterality in both upper and lower limbs. The physical demands of badminton suggest that injuries to the lower limbs may be a frequent occurrence [1], where the incidences are higher in the dominant lower limb due to the one-sided dominance [2]. In addition, habitual loading activities provoke changes in structural and mechanical properties of athletes’ tendons and muscles [3, 4]. Therefore, the prolonged practice of badminton added to the loading characteristics of the sport on the lower limbs could be one of the reasons for overuse injuries being approximately three times more frequent than traumatic injuries [2]. The most severe overuse injuries are related to tendons [1], including Achilles and patellar tendinopathies [1, 5]. Many investigations have studied the incidence of badminton injuries [1, 2, 6, 7] but we have not found any research that has analyzed chronic adaptations of the myotendinous structures to the continued practice of badminton as has been carried out in other sports [4].

Explosive and repetitive loading activities, such as lunges, are common in badminton and are related to a high risk of overuse injuries, such as patellar and Achilles tendinopathies [2, 5, 8–10]. Furthermore, increases in tendon size have been reported following training [11, 12], although equivocal evidence is available, with no changes [13] or changes only in mechanical properties also being reported [14–16]. Therefore, human tendon tissue can respond to single and repetitive mechanical loading bouts [6, 17, 18], which results in an increase in the cross-sectional area (CSA) of the tendon [19, 20]. Badminton involves repeated rapid forward lunges, especially on the dominant lower limb that can generate a load increase in the dominant compared to the non-dominant lower limb [21]. Nonetheless the difference in tendon structures between both lower limbs in badminton players has scarcely been studied. The studies carried out with badminton players showed a higher cross-sectional area (CSA) in the dominant patellar tendon compared to the non-dominant tendon as a consequence of the asymmetric loading during the game [20], and even greater intratendinous flow after the matches [8]. These results must be confirmed with further research since the number of participants in previous research was very limited [20] and the studies of the Achilles tendon in badminton players are also quite limited [8, 22].

The variables commonly used to describe muscle architecture are the fascicle length, pennation angle and CSA [23, 24]. According to previous studies [23, 24] these parameters provide insight into muscle function and are among the most important inputs to musculoskeletal models. Asymmetric sport-specific demands, may result in bilateral imbalances between dominant and non-dominant lower limb muscle architecture [25] which could provoke asymmetric results in strength tests [26, 27], with the highest differences being found in unilateral sport modalities [28]. Furthermore, eccentric training programs have shown significant increases in muscle thickness [29, 30], although, controversial results were described in relation to fascicle length and pennation angle adaptations [29–31]. The eccentric component of lunges [21], could cause greater thickness of the muscle architecture in the dominant lower limb in comparison with the non-dominant one in badminton players. Finally, muscle architecture is related to sport performance, and the muscle adaptations to exercise have been described in sports such as athletics, where fascicle length of the vastus lateralis showed a significant correlation with time in the 100 meters sprint [32]. However, the changes in muscle architecture due to badminton practice and their relation to sport performance have barely been studied.

The mechanical properties of muscles and tendons have been related to dynamic performance [33] and the greater stiffness values are beneficial for fast stretch shortening cycle (SSC) activities and actions involving high movement velocity [33]. According to previous studies, the asymmetric loading profiles between dominant and non-dominant lower limbs may cause differences in mechanical tendon properties, such as higher Achilles tendon stiffness on the dominant side [34], although there is greater controversy regarding muscle adaptations [29, 30]. Nonetheless, the effect of lower limb dominance on muscle and tendons stiffness in badminton players has not been evaluated in previous studies, where only the differences between injured and healthy patellar tendons have been explained [6]. Myotonometry is a new non-operator-dependent technology used to assess the stiffness of lower limbs [35] which requires less experience from the operator than other techniques, such as ultrasonography [35]. In addition, the feasibility, reproducibility and validity of using myotonometry in the assessment of myotendinous stiffness have been previously demonstrated [35, 36]. However, there are only a few studies that have reported myotonometry assessment of muscles and tendons in sport [36, 37], but none was made with badminton players.

Badminton is a racquet sport in which the prevalence of one side over the other could cause chronic asymmetries derived from continued practice, such as higher CSA and stiffness values in dominant lower limb tendons. Despite the popularity of the sport and the importance of knowledge of the effect of practice on myotendinous structures and mechanical properties, there are not many studies which have studied it [5, 6, 8]. Therefore, the main purpose was to determine whether asymmetry between the preferred and non-preferred lower limbs is present in the lower limb tendon structure, muscle architecture and stiffness values of professional badminton players.

## Methods

### Participants

Sixteen male professional badminton players (age = 24.1 ± 6.7 years; height = 177.90 ± 7.53 cm) participated in this study. All the players were ranked in the top 100 of the Badminton World Federation and they qualified for the 2017 World Championship. Sample size was previously calculated based on Couppe et al. [6] who measured patellar tendon cross-sectional area in elite male badminton players to analyze the differences between the lead extremity (146 ± 18 mm^2^) compared with the non-lead extremity (128 ± 12 mm^2^). We employed the following formula to calculate sample size: $n=\frac{2∙{\left(Z_{\propto /2}+Z_{1-\beta }\right)}^{2}∙\sigma^{2}}{d^{2}}$ [38]. Where *n* is the minimal number of subjects, Z_α/2_ is the normal deviate for a type I error (for 5% level of significance; Z_α/2_ = 1.96), Z_1-β_ is the normal deviate for the power of the study (for 90% statistical power; Z_1-β_ = 1.28), σ is the pooled standard deviation and *d* is the difference of means. The minimal number of subjects required to attain a power of 0.9 and a bilateral alpha level of 0.05 was calculated to be 15. The players included in the study were previously registered at the High Performance Training Centre in Spain to complete two weeks of training before the World Championship. High Level badminton players are individuals selected by their national federation for training specific performance criteria. All players from the High Performance training group were eligible and invited to participate. Players with an injury or any pain that would prevent them from doing their usual sport practice and also those players who had suffered an injury in the previous two years or a surgical operation such as Achilles tendon surgery anytime were excluded from the sample. All participants read and signed an informed consent, according to the declaration of Helsinki and were asked to identify their dominant hand. This study was approved by the Medical Ethical Committee of the Virgen de la Salud Hospital that served as a central committee for this project.

### Experimental design

The investigation was a descriptive study where two tests were carried out to assess the structural and mechanical properties of the vastus lateralis (VL), medial gastrocnemius (MG) and lateral gastrocnemius (LG) muscles, and the patellar (PT) and Achilles (AT) tendons. All structures were evaluated in the dominant and non-dominant lower limb. Ultrasound analysis was employed to measure the structural variables, and the mechanical properties were measured with a hand-held MyotonPro.

The following descriptive variables were recorded: age (years) and height (cm). The dependent variables analyzed in muscle structure were: fascicle length (cm), pennation angle (^o^), muscle thickness (cm) and fat layer (cm). The dependent variables analyzed in tendon structure were: thickness (cm), width (cm), CSA (cm^2^) and fat layer (cm). The dependent variables analyzed in the myotonometry test of muscles and tendons were: tone (Hz), logarithmic decrement (A.U.) and stiffness (N*m^-1^). The independent variable was established as the dominant lower limb vs. the non-dominant lower limb.

### Muscle architecture and tendon dimensions

**Fig 1:**
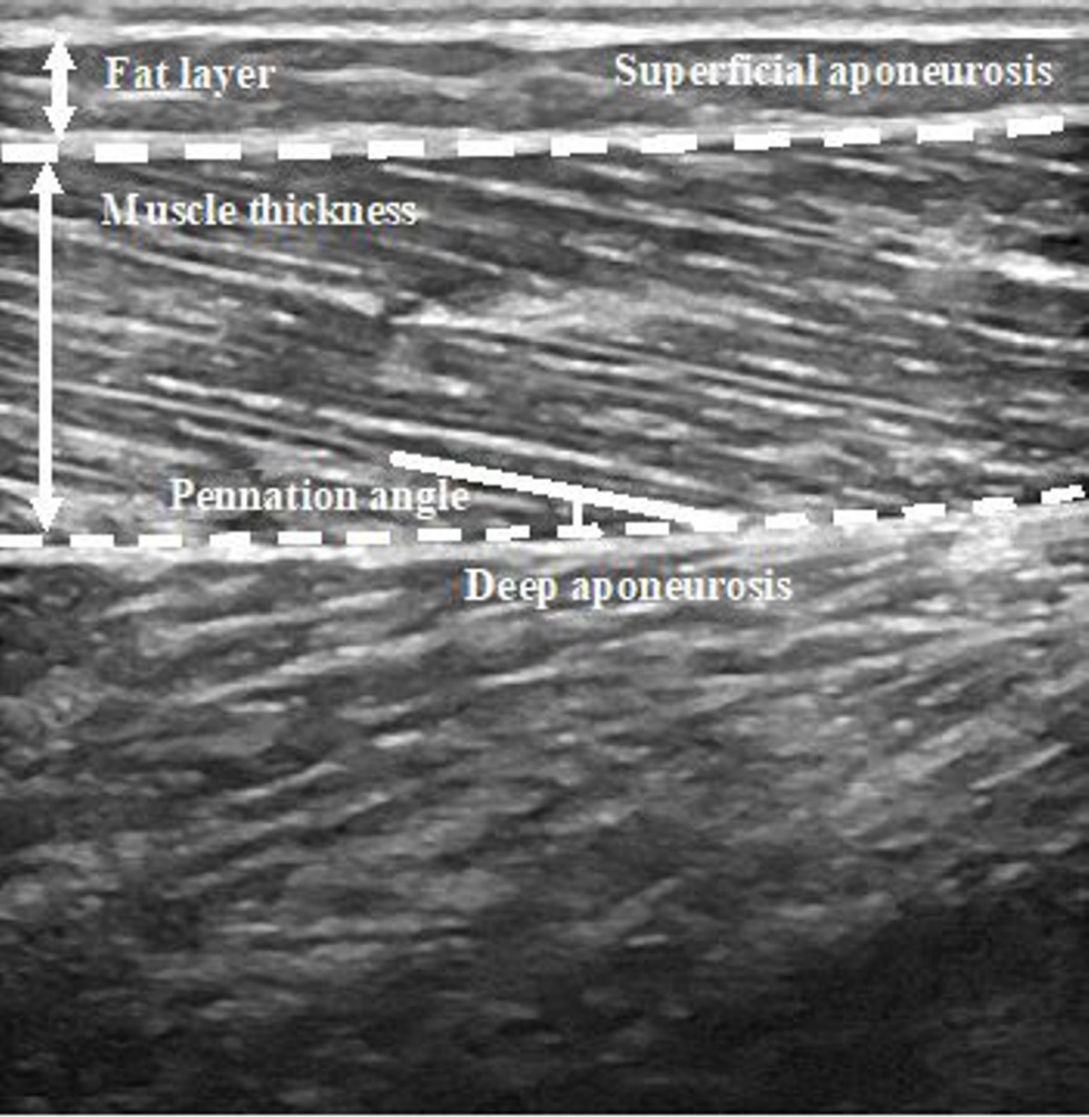
Example of fascicle tracking on an ultrasound image. Muscle thickness was calculated as the distance between the deep and superficial aponeurosis. Pennation angle was calculated as the angle between the deep aponeurosis and the tracked fascicle.

An experienced sports traumatologist with extensive musculoskeletal ultrasound training (JFJ), who was blinded to the clinical history, carried out all ultrasound examinations. A viscous ultrasound scanning gel was used as an interface, and was not warmed. All muscles and tendons were examined with high-resolution grey scale ultrasound and with B-mode, Logiq V2 (General Electric, Wauwatosa, WI). A measurement site was located longitudinally at: 50% of thigh length measured from the most prominent point of the greater trochanter to the lateral condyle for VL; 30% of leg length measured from the popliteal fossa to the lateral malleolus to GM and GL [25, 39]. The ultrasound probe was aligned with the fascicle direction to measure thickness and pennation angle of VL, GM and GL (Fig 1). Fascicle length across the deep and superficial aponeuroses was estimated using muscle thickness and pennation angle. Previously, this method of determining fascicle length has had a reported estimated coefficient of variation of 4.7% [40]. Fascicle length was calculated using the following equation: *Fascicle length = MT ∙ sinPNG*^−1^; MT represents the muscle thickness and PNG represents the pennation angle of each muscle.

Patellar and Achilles tendons were scanned in the sagittal and axial planes, taking care to avoid anisotropy. The thickness of the tendon was measured at 1 or 3 cm above the insertion of the patellar and Achilles tendon respectively and also at the insertion on the patella and calcaneus. These measurements have previously shown acceptable reliability [41]. Furthermore, the CSA of both tendons was measured at the same points on the axial plane.

The measurements were always taken with the players lying down. The VL and patellar tendons were scanned with the player in a supine position and the knee flexed at 15^o^ with a pillow. The MG, LG and Achilles tendons were scanned with the players placed prone and the feet hanging freely in a relaxed slightly plantar flexed position. All images were analyzed with Image analysis software (Image J, 1.47v, National Institute of Health, Maryland, USA) to measure fascicle length, pennation angle, muscle thickness, tendon thickness, tendon width, CSA of the tendon and fat layer.

### Measurement of muscle and tendon mechanical properties using MyotonPRO

MyotonPRO measures the deformation properties of natural damped oscillations produced following a brief (15 ms) mechanical tap on the surface of the skin [42]. Computational software creates data on elasticity (logarithmic decrement), tone (frequency) and stiffness of the underlying muscle, based on equations calculated from the acceleration of the testing probe during oscillations [43]. Elasticity is the biomechanical property of the muscle that characterizes the ability to recover its initial shape after a contraction or the removal of an external force [43]. Oscillation frequency shows the tone of the muscle in its testing state, which can be at rest without any voluntary contraction or contracted [43]. Stiffness is the biomechanical property of the muscle that explains the resistance of the muscle to a contraction or to an external force that deforms its initial shape [43]. All MyotonPro measurements were made with the device held perpendicular to the skin on the region of interest to reduce the effect of gravity on the properties of muscle and tendon tissues and get the highest effectivity of oscillations during the test [42].

### Statistical analysis

The statistical analysis was performed with IBM SPSS Statistics 23.0 (SPSS, Chicago, Illinois). All data are expressed as means ± standard deviation. The data were tested for normality with a Kolmogorov–Smirnov test. Since the assumption of normality (all variables P>0.05) was verified, overall significance of differences between the dominant and non-dominant lower limbs was determined using Student’s t test for related samples. Effect size statistics were used to quantify the magnitude of difference between the lower limbs, according to the formula proposed by Cohen [44]. The magnitude of the effect size was interpreted using the scale of Cohen [44]: an effect size lower than 0.2 was considered as small, an effect size around 0.5 was considered as medium and an effect size over 0.8 was considered as large. The relationship between structural and mechanical variables was analyzed with a simple linear regression, from which the Pearson correlation coefficient was calculated. A probability level p <0.05 was defined as statistically significant. Between-side comparisons (percentage difference calculated as: $100\%-\left([\frac{nondominant}{dominant}]∙100\%\right)$; were used as a measure of symmetry [42, 45]. For Myoton variables, a difference of up to 5% in the same muscle between two sides as measured by Myoton is still considered symmetrical [45].

## Results

### Muscle architecture

No difference was found in fascicle length, pennation angle and muscle thickness between the dominant and non-dominant lower limbs in VL (p = 0.894), MG (p = 0.184) and LG (p = 0.392), nor in the fat layer of any of them (p = 0.560). (Table 1).

**Table 1:** Muscle architecture variables.

	Dominant	Non-Dominant	Δ (%)	95% CI	Effect Size
Vastus Lateralis					
Thickness (cm)	2.08 ± 0.40	2.07 ± 0.44	0.41	- 0.13 to 0.15	< 0.1
Pennation angle (^o^)	14.66 ± 3.26	14.53 ± 2.69	0.85	- 2.08 to 2.33	< 0.1
Fascicle length (cm)	8.46 ± 1.81	8.51 ± 2.21	- 0.58	- 1.50 to 1.41	< 0.1
Fat layer thickness (cm)	0.51 ± 0.30	0.52 ± 0.35	- 0.58	- 1.50 to 1.41	< 0.1
Medial Gastrocnemius					
Thickness (cm)	1.53 ± 0.34	1.61 ± 0.28	- 4.85	- 0.19 to 0.04	0.3
Pennation angle (^o^)	24.38 ± 5.46	24.36 ± 6.05	0.17	- 3.28 to 3.36	< 0.1
Fascicle length (cm)	3.78 ± 0.67	4.09 ± 1.06	- 8.09	- 0.91 to 0.30	0.3
Fat layer thickness (cm)	0.43 ± 0.16	0.42 ± 0.19	3.87	- 0.04 to 0.08	0.1
Lateral Gastrocnemius					
Thickness (cm)	0.95 ± 0.40	0.91 ± 0.38	3.79	- 0.15 to 0.21	0.1
Pennation angle (^o^)	12.82 ± 3.40	11.97 ± 4.72	6.64	- 1.21 to 2.91	0.2
Fascicle length (cm)	4.38 ± 1.77	4.55 ± 1.57	-3.97	- 1.34 to 0.99	0.1
Fat layer thickness (cm)	0.35 ± 0.13	0.36 ± 0.18	- 2.65	- 0.05 to 0.03	0.1

CI = Confidence Interval

### Tendon structure

**Fig 2:**
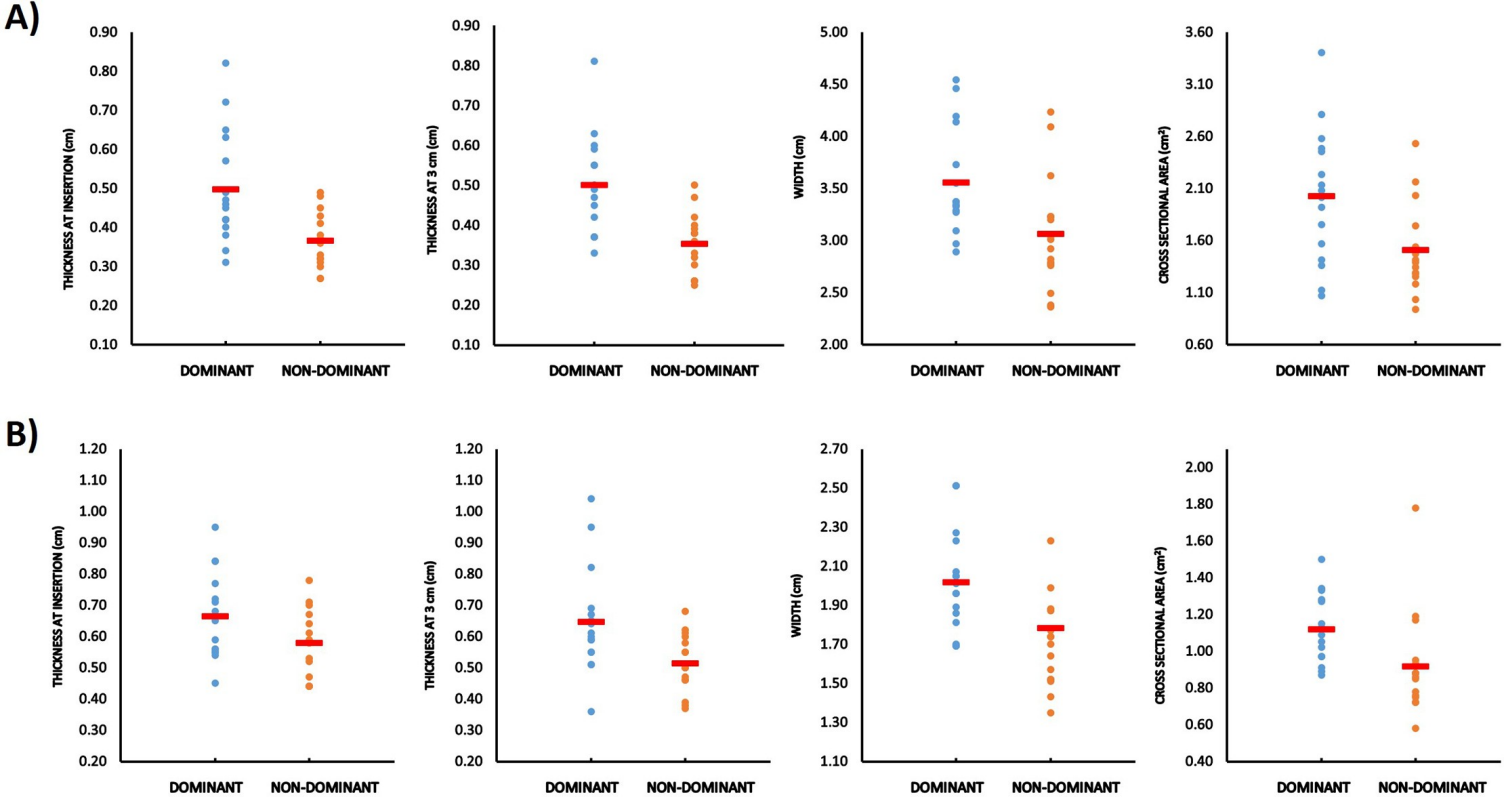
Significant differences of structural variables of patellar and Achilles tendons. A) Results of patellar tendon. B) Results of Achilles tendon. Individual Values and means.

Bilateral differences were observed in the patellar and Achilles tendon (Fig 2). The thickness of the patellar tendon (dominant vs. non-dominant) was 0.13 ± 0.16 cm greater for the dominant lower limb at the insertion point (0.50 ± 0.14 cm vs. 0.37 ± 0.07 cm; p = 0.007; ES = 1.2) and 0.15 ± 0.13 cm at 1 cm distance from the patella (0.50 ± 0.12 cm vs. 0.35 ± 0.08 cm; p < 0.001; ES = 1.5). The patellar tendon width was 0.50 ± 0.65 cm (3.55 ± 0.51 cm vs. 3.06 ± 0.54 cm; p = 0.008; ES = 0.9), CSA 0.52 ± 0.60 cm^2^ (2.02 ± 0.64 cm^2^ vs. 1.51 ± 0.42 cm^2^; p = 0.004; ES = 0.9) and fat layer 0.12 ± 0.15 cm (0.63 ± 0.15 cm vs. 0.51 ± 0.13 cm; p = 0.004; ES = 0.9) greater in the dominant lower limb of the badminton players.

Similar results were observed in the Achilles tendon (dominant vs non-dominant), the thickness was 0.08 ± 0.12 cm greater (0.66 ± 0.14 vs. 0.58 ± 0.10; p = 0.032; ES = 0.7) at the insertion point and 0.13 ± 0.21 cm greater (0.65 ± 0.17 vs. 0.51 ± 0.09; p = 0.022; ES = 1.0) at 3 cm from the insertion point for the dominant compared to the non-dominant lower limb. In addition, the CSA was 0.20 ± 0.26 cm^2^ (1.12 ± 0.18 vs. 0.92 ± 0.28; p = 0.007; ES = 0.8) and the width 0.23 ± 0.38 cm greater (2.02 ± 0.26 vs. 1.78 ± 0.40; p = 0.027; ES = 0.7) in the dominant leg. No significant differences were found in the fat layer between the lower limbs, dominant and non-dominant (p = 0.655).

### Myoton values

The values obtained for the decrement, frequency and stiffness in the VL, MG, LG, patellar and Achilles tendon are shown in Table 2. No significant differences were found between the dominant and non-dominant side for any of the variables studied.

**Table 2:** Myometric data of muscles and tendons.

	Dominant	Non-Dominant	Δ (%)	95% CI	Effect size
Vastus Lateralis					
Decrement (A.U)	1.37 ± 0.20	1.34 ± 0.24	2.07	- 0.05 to 0.11	0.1
Frequency (Hz)	16.68 ± 3.08	16.50 ± 3.19	0.57	- 0.97 to 1.32	0.1
Stiffness (N*m^-1^)	316.69 ± 50.30	316.75 ± 74.49	0.06	- 29.47 to 29.35	< 0.1
Medial Gastrocnemius					
Decrement (A.U.)	1.18 ± 0.19	1.16 ± 0.17	1.68	- 0.03 to 0.09	0.1
Frequency (Hz)	15.84 ± 1.71	15.44 ± 1.27	2.12	- 0.15 to 0.95	0.3
Stiffness (N*m^-1^)	273.94 ± 29.62	264.06 ± 20.67	3.14	- 0.58 to 20.33	0.4
Lateral Gastrocnemius					
Decrement (A.U.)	1.16 ± 0.18	1.17 ± 0.21	- 0.76	- 0.07 to 0.06	0.1
Frequency (Hz)	16.52 ± 1.48	16.76 ± 2.07	- 1.38	- 0.81 to 0.31	0.1
Stiffness (N*m^-1^)	299.94 ± 30.54	303.13 ± 50.28	- 0.74	- 18.46 to 12.09	0.1
Patellar tendon					
Decrement (A.U.)	0.88 ± 0.11	0.90 ± 0.15	- 3.12	- 0.09 to 0.04	0.2
Frequency (Hz)	20.45 ± 4.19	20.21 ± 3.51	0.36	- 0.61 to 1.09	0.1
Stiffness (N*m^-1^)	515.13 ± 180.50	518.63 ± 168.73	- 4.01	- 46.73 to 39.73	< 0.1
Achilles tendon					
Decrement (A.U.)	0.90 ± 0.22	0.96 ± 0.31	- 5.54	- 0.16 to 0.06	0.2
Frequency (Hz)	29.86 ± 2.07	29.42 ± 2.07	1.31	- 0.50 to 1.37	0.2
Stiffness (N*m^-1^)	750.00 ± 71.45	741.25 ± 70.06	1.03	- 12.11 to 29.61	0.1

CI = Confidence Interval

A negative correlation was found between the fat layer and the stiffness value in the VL (r = -0.729; p = 0.001), MG (r = -0.459; p = 0.011), LG (r = -0.424; p = 0.019) and patellar tendon (r = -0.417; p = 0.022).

## Discussion

The purpose of the present study was to investigate chronic adaptations in muscle architecture and tendon structure caused by the prolonged practice of badminton and to document possible differences between dominant and non-dominant lower limbs. Our findings show no bilateral differences in the muscle architecture of the VL, MG and LG, and nor in the myometric variables of the muscles and tendons analyzed. On the other hand, asymmetries were observed between the dominant and the non-dominant lower limbs in the tendon structure for the patellar and Achilles tendons. The dominant lower limb showed greater values of thickness, CSA and width than the non-dominant lower limb in both patellar and Achilles tendons. Taking this into account, it would be advisable to carry out a compensatory training program to prevent overuse injuries that affect the tendons of the dominant lower limb, which are some of the most common injuries in badminton [5, 7, 10].

The present data showed a tendon hypertrophy response associated with chronic loading as evidenced by the greater tendon CSA (16–26%) of the patellar tendon in the dominant lower limb, similar to the results obtained by other researchers that evaluated the patellar tendon in badminton players [6]. Patellar tendons can withstand very high forces during lunge movements, which are some of the most repeated actions during badminton matches [21]. It has been estimated that forces within the patellar tendon may reach 8000–11100 N during running and jumping activities [46], which means that the tendon can be subjected to stresses in excess of 100 MPa (assuming a CSA of 1 cm^2^). It has previously been shown that the habitual loading of the dominant lower limb in badminton players generates a side-specific increase in patellar tendon CSA, and the present data from badminton players confirm these findings [20]. The tendon CSA value is not only important when describing the adaptation of the tendon to exercise [19] but it is also one of the variables studied during the diagnosis of injuries associated with other variables such as pain [6]. A small CSA associated with overuse of the tendon during exercise may be one of the triggers of tendinopathy [6].

Tendinopathy is a major problem among athletes performing explosive extensions of the knee and repetitive loading activities involving rapid forward lunges, as seen in several sports, for example, badminton [7]. These injuries mainly affect the patellar and Achilles tendons [7, 8]. The Achilles tendon has been less studied from the point of view of the tendinous structure in badminton players [22], as most of the research has focused on describing the intratendinous blood supply associated with pain [9]. Therefore, the results obtained in our research help us to ascertain the adaptation of the Achilles tendon to the practice of badminton. As in the patellar tendon, we observed higher variable values in the Achilles tendon of the dominant lower limb compared with the non-dominant one. The adaptation of the Achilles tendon to exercise has already been described in sports such as running in which significant differences were found between CSA values in runners and non-runners, with higher values in athletes [47]. The one-sidedness of badminton does generate asymmetric adaptations to exercise in the Achilles tendon variables, with higher values in the dominant side for CSA, thickness and width as described in previous studies for the patellar tendon [20].

No differences were found in the variables related to muscle architecture between the dominant and non-dominant lower limbs, contrary to what is described by other authors who evaluated unilateral sports [25]. Previous research suggests that the preferential use of the dominant over the non-dominant lower limb is associated with a larger muscle mass in the dominant one [48]. In addition, unilateral sports with a high volume of strong eccentric movements of the dominant limb would appear to cause an increase in the length of the muscle fascicle and a reduction of pennation angles [49] that have been described as asymmetric changes in modalities such as snowboarding and soccer [25, 50]. Contrary to these findings, in the work of Aeles et al. [51] involving well-trained jumping athletes and untrained subjects, no significant differences were found in muscle architecture between the dominant and non-dominant lower limbs, and additionally some of the most recent reviews indicate that muscle architecture could be unrelated to achieving an enhanced sport performance in jumping [51, 52]. Therefore, changes in muscle architecture in trained athletes would not show so many chronic adaptations to exercise but would represent acute changes resulting from muscle fatigue caused by the activity and would disappear after 15 minutes of recovery [53, 54]. Another possible reason why we do not find asymmetries in the muscle architecture variables is that the movements are symmetrical, except at the end when the dominant lower limb is the one that performs actions like lunges affecting the tendon that is more in charge of supporting the tension of the eccentric movements [21].

Chronic adaptation to exercise has also been measured in the quality of different tissues such as stiffness, measured with a hand-held Myoton and used to describe tendons and muscles [36, 43, 55]. Higher levels of lower-body stiffness appear to be advantageous when performing rapid and repeated SSC movements [33], which are common during badminton matches [21]. The values shown by the badminton players are similar to those found in the evaluation of athletes of other modalities such as football, although we did not find any significant differences derived from the one-sidedness of the sport, something that had been described in physically active people when evaluating the Achilles tendon [34]. One of the reasons may be that stiffness measurements are influenced by the superficial fat layer of muscles and tendons. The correlations obtained in our study showed a negative relationship between the surface fat layer and the stiffness value, except for the Achilles tendon in which fat layer values were smaller than at the rest of the measurement points. Previous studies showed lower to moderate correlations between fat layer values and MyotonPro measurements [56, 57] so more investigations are necessary in order to clarify the influence that the fat layer could have on the results. In addition, previous studies, which compared Myoton Pro with shear wave elastography, showed a high correlation between both methods [58, 59]. Therefore, Myoton Pro is a reliable instrument to assess the stiffness of superficial tissues, with less lower operator dependency than ultrasound techniques [58, 59].

## Conclusions

No differences between dominant and non-dominant lower limbs were found in the muscle architecture of the vastus lateralis, medial gastrocnemius and lateral gastrocnemius or in the myometric variables of the muscles and tendons analyzed. On the other hand, the prolonged practice of badminton caused asymmetries in the morphologic structure of the patellar and Achilles tendons. The dominant lower limb showed greater values in thickness, CSA and width of the patellar and Achilles tendons than the non-dominant lower limb. In addition, the negative correlation between the stiffness and the fat layer makes it necessary to take the fat layer into account in the evaluation of athletes when using MyotonPro technology.

## Supporting information

S1 Dataset (XLSX)
